# *Galleria mellonella* model recapitulates *Staphylococcus aureus* hemolysin toxicity and antibody-mediated mechanisms of protection

**DOI:** 10.3389/fmicb.2026.1809973

**Published:** 2026-05-14

**Authors:** Edoardo Cherubini, Andrea Paola Mandelli, Lucia Henrici De Angelis, Michela Brazzoli, Marco Maria D’Andrea, Fabio Bagnoli, Silvia Rossi Paccani, Emiliano Chiarot

**Affiliations:** 1 GSK, Siena, Italy; 2 Department of Biology, University of Rome Tor Vergata, Rome, Italy

**Keywords:** alpha-hemolysin, antimicrobial resistance, host-pathogen interactions, monoclonal antibodies, *Staphylococcus aureus*

## Abstract

The study of host-pathogen interactions in animal infection models has clarified mechanisms of bacterial pathogenicity and supported the development of preventive and therapeutic strategies. Although many virulence factors have been identified *in vivo* using mammals such as mice and rats, this work requires large numbers of animals. Over the past two decades, insect-based alternatives have gained popularity due to ethical and practical advantages. This study evaluated *Galleria mellonella* larvae as an alternative *in vivo* model for investigating microbial pathogenicity, focusing on *Staphylococcus aureus* and its conserved toxin alpha-haemolysin (Hla). Larvae were injected with purified Hla or infected with bacteria, and survival, clinical scores, and bacterial burden were monitored. Results showed that larvae are susceptible to Hla-mediated toxicity, whether the toxin is administered directly or produced during infection. Notably, pre-treatment with a human anti-Hla monoclonal antibody protected larvae from toxin-induced disease and staphylococcal infection, similarly to what is reported in more extensively studied mammal models. These findings support *Galleria mellonella* as a cost-effective, ethical model for studying pathogenicity, characterizing virulence factors, and evaluating targeted treatments such as monoclonal antibodies. When combined with high-throughput *in vitro* assays, this approach may accelerate the discovery of new therapeutic and preventive interventions while reducing reliance on mammalian models.

## Introduction

The study of host-pathogen interactions in animal models of disease has proven essential for understanding the mechanisms that pathogens use to overcome the host immune system and, conversely, the countermeasures employed by the immune system to prevent bacteria from gaining the upper hand (Akira et al., 2006). This knowledge has guided the development of preventive and therapeutic strategies that have helped humanity to fight infections once responsible for millions of deaths before these interventions were introduced (Hunter et al., 1973; Mishra et al., 2025). Thanks to the increasing knowledge of pathogenic mechanisms, the host immune system’s modes of action, and the advent of technologies suitable for preclinical studies, animal models that can virtually represent most human infections—especially those using rodents—have been developed over the last century (Rong and Liu, 2023). These models have taken advantage of bacteria’s ability to infect different hosts and the similarities among mammalian immune systems, and they were proven to be sufficiently translatable to justify their use (Bailey et al., 2013; Medetgul-Ernar and Davis, 2022). Nevertheless, with the growing sensitivity toward the use of animals for experimental purposes and the complexity of performing experiments with mammals, the importance of developing alternative animal models that do not use mammals has become increasingly evident in recent years (Bailone et al., 2020; Chiarot and Pizza, 2022).

Several animal models of infection have been developed in the last two decades using invertebrates, including insects and worms which have demonstrated the ability to recapitulate some findings already observed in mammalian hosts (Borman, 2018; Serrano et al., 2023). Although these models are often not suitable for addressing questions about the host immune system that are directly translatable to humans, they can offer the opportunity to study virulence factors and, for example, to test preventive or therapeutic interventions targeted against those factors (Tan et al., 1999; Flores et al., 2020; Durieux et al., 2021). Among the explored hosts, larvae of the greater wax moth *Galleria mellonella* (*G. mellonella*) have received particular attention in recent decades due to their unique features (Piatek et al., 2021). They are easy to handle, very inexpensive, and possess a simple yet functional immune system (Serrano et al., 2023; Gallorini et al., 2024). On this basis, *G. mellonella* has been extensively used to assess infectivity and study potential treatments against almost all human pathogens, including viruses, bacteria, and fungi (Younghusband and Lee, 1969; Asai et al., 2019; Ménard et al., 2021; Marena et al., 2025). Interestingly, several recent works have employed this model without confirming results in mammalian models, underscoring the growing perception of its reliability within the scientific community (Seed and Dennis, 2009; Ramalho et al., 2022).

The path from an “alternative model” to a “validated model” however, still requires additional confirmatory studies addressing several important questions, such as: (i) can the model be useful to test virulence factors responsible for human diseases? (ii) is it suitable for assessing the efficacy of treatments specifically targeted against these virulence factors?

On this ground, we selected the human pathogen *Staphylococcus aureus* (*S. aureus*) and its well-characterized virulence factor, alpha-hemolysin (Hla), to further investigate this host model. Our choice was mainly driven by the fact that *S. aureus* has already been shown to be infective in *G. mellonella* and that several readouts (e.g. survival, clinical signs, and bacterial burden) could be used as endpoints (Seed and Dennis, 2009; Li et al., 2020; Menard et al., 2021). Additionally, Hla is a secreted toxin commonly produced by *S. aureus* clinical strains that is expected to exert its toxicity in larvae of *G. mellonella* (Chen et al., 2021; Li et al., 2023; Guo et al., 2024; Hao et al., 2024; Rodrigues et al., 2024). Having identified an analog of ADAM10, the Hla receptor in mammalian cells, in *G. mellonella*, we demonstrated that Hla is a key virulence factor for this model, and that a specific anti-Hla human monoclonal antibody (mAb) can effectively neutralize its toxicity *in vivo* (Wilke and Bubeck Wardenburg, 2010).

Data reported in the present work underscore the potential relevance of this invertebrate as a translatable host model for human pathogens. Interestingly, we also have demonstrated that this model is suitable for assessing new antibacterial treatments, such as monoclonal antibodies or other antigen-specific therapies. The preclinical selection of antimicrobial compounds might benefit from combining high-throughput *in vitro* and *in vivo* analyses (like the one we are proposing here) before embarking on more classical, yet challenging, *in vivo* studies, thereby increasing the likelihood of finding new treatments for unmet clinical needs by using a more ethical and straightforward approach.

## Materials and methods

### Larvae procurement and thermal acclimation

Larvae of *G. mellonella* were purchased from two commercial suppliers (SA.GI.P. S.a.s. or Insectfarm S.r.l., Italy). Upon arrival, larvae were maintained at 15 °C until use. On the day of the experiment, larvae were removed from 15 °C storage and placed at room temperature for 90 min to acclimate. Dead larvae were discarded, and then those selected had a body weight within the range of 0.38–0.52 g, with no melanization spots or lymph clots. Animals were then randomized into experimental groups—each comprising 6–12 animals, depending on the experiment—and maintained in sterile Petri dishes at 37 °C with 5% CO_2_ for 30 ± 5 min for acclimation. In all experiments, an untreated group (vitality) and a mock-treated group (PBS) were included. However, data from the vitality group were not visualized in the figures, as it served solely to confirm the overall viability and suitability of the larvae for experimentation.

### Bacterial strains and preparation of the inoculum

*S. aureus* USA300 LAC, either wild-type (WT) or Hla knockout (ΔHla) strains, were used for the staphylococcal infection model. Strains were kindly provided by the University of Chicago (Chicago, Illinois, United States). Frozen stocks of bacteria were prepared by initially thawing an existing stock and growing the bacteria in tryptic soy broth (TSB) at 37 °C + 5% CO_2_ with agitation for 2.5 h, until the early exponential phase (OD_600_ ∼ 2.0, corresponding to approximately 1.0–2.0 × 10^9^ CFU/mL). Bacteria were then diluted 1:1 in a freezing solution composed of phosphate-buffered saline (PBS, Gibco), 1% bovine serum albumin (BSA, Sigma-Aldrich), and 10% L-glutamic acid (Sigma-Aldrich). Aliquots were stored at -80 °C in cryovials until use. For inoculum preparation, frozen stocks of bacteria were diluted 1:25 in fresh TSB and incubated at 37 °C + 5% CO_2_ with agitation until the bacteria reached the early exponential phase, then pelleted, washed once in sterile 1X PBS, and diluted to the desired concentration.

### Treatment of larvae with toxin, bacteria, and/or monoclonal antibodies

Before injection, selected larvae were removed from the incubator, and their abdomens were disinfected with 70% ethanol. Animals were injected (10 μL inoculum) intrahemocoelically into the last right proleg using a Hamilton syringe with a 30-gauge needle (Hamilton Company, Reno, NV, United States) and held still for a few seconds after injection to allow peristalsis to evenly distribute the treatment. When the mAb was administered before the toxin or bacteria challenge, the second injection was performed 4 h ± 30 min after the first one, in the opposite proleg. After the treatment, larvae were placed in Petri dishes lined with paper and food. Larvae were kept at 37 °C and 5% CO_2_ throughout the observation period and activity, melanization, and survival were recorded and evaluated using the clinical scoring system described above (Table 1). For some experiments, larvae were sacrificed the day after the infection and homogenized in sterile PBS using a tissue homogenizer (GentleMACS Tissue Dissociator), following the manufacturer’s instructions. The resulting suspension was then serially diluted and plated on mannitol salt agar plates to assess the bacterial burden (expressed as CFU/larva). Mannitol salt agar was chosen as a selective medium, because it inhibited the growth of the natural microbial population of *G. mellonella*.

**TABLE 1:** Clinical scoresheet for *G. mellonella* larvae.

Melanization		Activity	
0	No melanization	0	Normal
1	Minimal melanization^1^	1	Minimal reduction
2	Diffused melanization	2	Severe reduction^2^
3	Dead larva	3	Dead larva

^1^Cloth(s) and/or grey/dark brown colored.

^2^Reduced response to physical stimuli.

### Survival and clinical score

After each treatment, larvae were closely monitored daily throughout the study, unless otherwise stated. For survival data, dead larvae (defined as unresponsive to stimuli and completely melanized) were recorded and removed from the experimental group. Animals that survived until the end of the observation period were euthanized per protocol and were right-censored at their final daily assessment, as euthanasia at the scheduled study endpoint did not constitute an event. In addition to survival data, clinical scores based on the presence and degree of melanization and on larval activity were assigned to each animal after the treatment in some experiments (Table 1). Individual scores were recorded daily (unless otherwise stated) and used to compute a daily total score, which was then cumulatively analyzed by calculating the area under the curve (AUC).

### Toxin and mAb purification

Hla toxin was cloned and purified as described elsewhere (Bagnoli et al., 2015). Briefly, Hla-expressing *Escherichia coli* cells were harvested and mechanically disrupted; recombinant proteins were recovered from the supernatant and purified by using a hydrophobic interaction chromatography (HIC) resin and analyzed for purity and integrity by SDS-PAGE, Size-Exclusion and Reversed-Phase High-Performance Liquid Chromatography (SE-HPLC, RP-HPLC).

Briefly, the anti-Hla mAb was expressed in ExpiCHO cell lines, derived from Chinese hamster ovary (CHO) cells, harvested and purified using MabSelect SuRe™ antibody purification resin following manufacturer’s instructions.

### Coupling of Hla toxin to magnetic Luminex beads

A total of 20 μg of recombinant Hla was chemically coupled to 1.25 × 10^6^ MagPlex beads (Luminex) using an automated coupling method on a liquid handling workstation (Hamilton-Microlab STAR IVD). Briefly, the antigen was coupled via a two-step carbodiimide procedure during which the microsphere carboxyl groups were first activated with 1-ethyl-3-(3-dimethylaminopropyl) carbodiimide hydrochloride (EDC, Pierce) in the presence of sulfo-NHS (Pierce) to form a sulfo-NHS ester intermediate. The reactive intermediate was then replaced by a reaction with the target molecule’s primary amine to form a covalent amide bond. The coupled beads were stored at 4 °C for up to 14 days.

### Analysis of antibody stability with Luminex assay

After treating larvae of *G. mellonella* with the anti-Hla mAb, animals were sacrificed at different time points and homogenized in 5 mL of PBS using a tissue homogenizer (GentleMACS Tissue Dissociator) following the manufacturer’s instructions. Following homogenization, each sample was diluted 1:100 in PBS and transferred in duplicate to a flat-bottom, non-binding 96-well plate, and eight three-fold serial dilutions were performed. Negative (PBS) and positive (7.4 mg/mL of anti-Hla mAb in PBS) controls were included in the assay. Samples were then incubated with Hla-coupled beads for 1 h at room temperature with agitation (800 rpm). After three washes with PBS containing 0.05% Tween-20, a goat anti-human IgG conjugated with phycoerythrin (Jackson ImmunoResearch, RRID:AB_2632442) was added, and the plates were incubated for 30 min at room temperature with agitation (800 rpm). Following this, the wells were washed again three times with PBS-Tween-20, and the beads were resuspended in PBS. The assay was read using a Luminex 200 reader set to a high RPI target with a timeout of 120 s. For each time point, the mAb recovery rate in larval homogenates was calculated as the percentage of the mAb titer recovered over time, relative to the median value obtained at time 0.

### Statistical analysis

Statistical analysis was performed using GraphPad Prism version 8.0.0 (GraphPad Software, Inc., La Jolla, United States). The nonparametric Mann-Whitney post *t* test was used to assess differences between two groups (e.g., when evaluating differences between the WT and ΔHla *S. aureus* USA300 strains and between isotype-treated and anti-Hla mAb-treated infected larvae). One-way ANOVA (Kruskal-Wallis) followed by uncorrected Dunn’s post-test was used to assess differences among three or more groups (e.g., in the molar ratio studies, the *S. aureus* USA300 LAC model setup). The log-rank (Mantel-Cox) and Gehan-Breslow-Wilcoxon tests were used to assess significance in survival rates (Kaplan-Meier curves). Finally, Fisher’s exact test was used to assess differences in frequencies (i.e., the number of live and dead larvae for Hla treatments). A *p* < 0.05 was considered significant for all analyses. AUC was calculated to perform a cumulative data analysis on clinical scores assigned to larvae throughout the studies. The trapezoidal AUC of the clinical scores for a single day was calculated using the following equation:


$$A\,U\,C_{0-1}=\frac{\left(B_{t\,0}+b_{t\,1}\right)\,\text{x}\,\left(t_{0}-t_{1}\right)}{2}$$

where *B*_*t*0_ stands for the longer base, *b*_*t*1_ for the shorter base, and *t* for time points. AUC was calculated for each time point after injection in each larva and then summed to obtain a final AUC value representing the entire period after intrahemocoelic injection.

## Results

### *S. aureus* Hla is toxic for larvae of *G. mellonella* and increases bacterial virulence

Animal models of infection developed in rodents have significantly contributed to study virulence mechanisms that lead to bacterial pathogenesis and to propose preventive and therapeutic antibacterial treatments targeting these mechanisms (Hunter et al., 1973; Sarkar and Heise, 2019). Here, we wondered whether a more ethical approach could have provided us with similar results and found that the combination of the invertebrate host model *G. mellonella* and the pathogen *S. aureus*, with its well-known and the ubiquitous secreted toxin Hla, might be the right choice to answer this question.

Indeed, Hla binds to the cellular receptor ADAM10, causing cell damage (Inoshima et al., 2011; Berube and Bubeck Wardenburg, 2013). Interestingly, *G. mellonella* potentially produces a protein annotated as “exposed disintegrin and metalloproteinase domain-containing protein 10 homologue” (XP_026755746.1) that shows high identity with human ADAM10 (41.4%), indicating that these larvae could be susceptible to the Hla toxin (Supplementary Figure 1). To test this hypothesis, we injected different doses of a recombinant form of Hla into larvae and monitored toxicity and survival for 7 days. As shown in Table 2, Hla induced clear, dose-dependent toxicity, with higher death rates at elevated concentrations.

**TABLE 2:** Hla is toxic for *G. mellonella* larvae within 7 days after inoculum.

Treatment	Treated larvae	Deceased larvae	Survived—severe signs^§^	Survived—mild signs^†^	Survived—no signs	Fisher’s exact *t*-test^#^
PBS	20	0	–	–	20	–
HT-Hla 10 μg/larva	10	0	–	1	9	ns (*p* = 1)
Hla 10 μg/larva	20	13	4	3	–	**** (*p* < 0.0001)
Hla 5 μg/larva	10	5	1	2	2	** (*p* = 0.0018)
Hla 2.5 μg/larva	10	4	1	1	4	** (*p* = 0.0077)

HT, Heat-treated. ^§^Severe signs: clinical score values between 4 and 5 (sum of activity and melanization scores).

† Mild signs: clinical score values between 1 and 3 (sum of activity and melanization scores).

^#^Number of survived larvae/number of deceased larvae, for Hla-treated larvae versus PBS-treated.

**p < 0.01, ****p < 0.0001.

**FIGURE 1:**
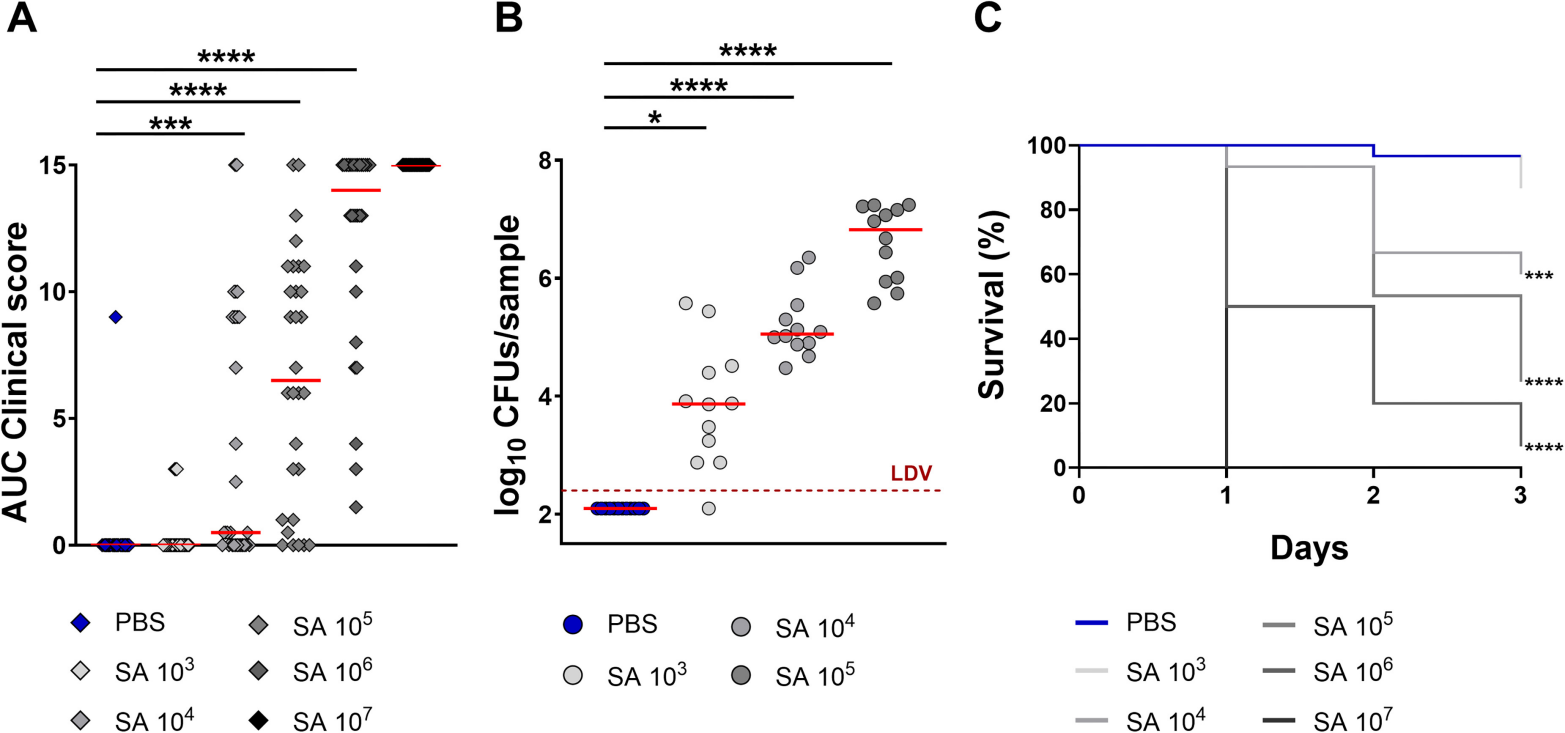
Infection with *S. aureus* USA300 LAC is lethal for larvae of *G. mellonella*. Clinical signs of disease, CFU counts at day one post infection in homogenized larvae and survival observation until 3 days after infection were all reliable readouts for the *in vivo* model. **(A)** AUC analysis of clinical scores assigned to larvae (*n* = 30 per group, three independent experiments) infected with different doses of the *S. aureus* USA300 LAC strain. Larvae were observed daily after infection and clinical scores for melanization and activity were recorded as described in the text. **(B)** Bacterial burden observed in larval homogenates, expressed as log_10_ CFUs/sample. Infected larvae (*n* = 12 per group, two independent experiments) were sacrificed the day after infection. LDV, lower detectable value. **(C)** Survival curves of larvae (*n* = 30 per group, three independent experiments) infected with different doses of *S. aureus* USA300 LAC. For **(A,B)**, each single dot reported data for one animal, while the solid red line was the median value of the group. Legend: *p < 0.05, ***p < 0.001, ****p < 0.0001, comparisons have been made between infected and mock-treated larvae. For **(A,B)**, the Kruskal-Wallis, uncorrected Dunn’s post-test was used to assess significance. For **(C)**, the log-rank (Mantel-Cox) and the Gehan-Breslow-Wilcoxon tests were used.

**FIGURE 2:**
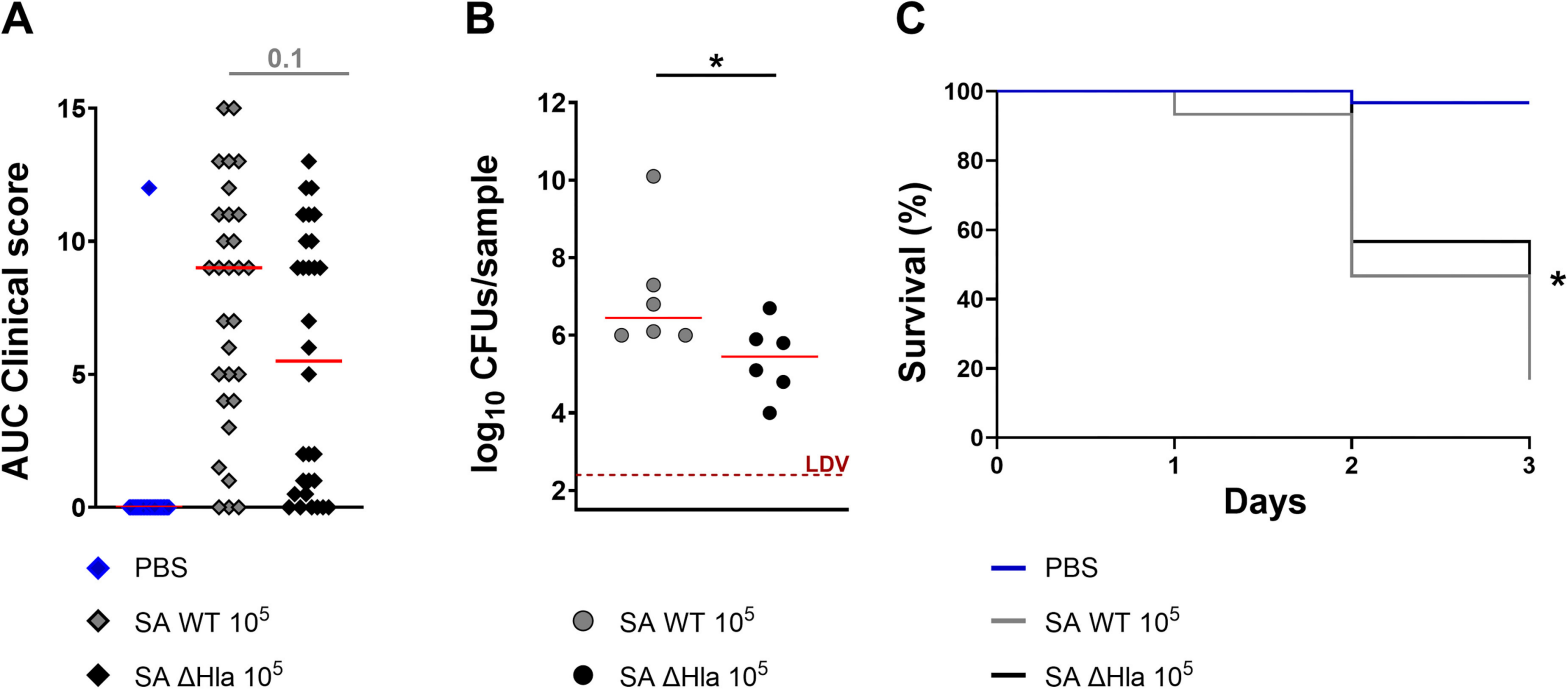
Hla enhances *S. aureus* virulence in larvae of *G. mellonella.* Comparison between the virulence of the wild type *S. aureus* USA300 LAC strain and that of its isogenic Hla mutant in larvae of *G. mellonella*. **(A)** AUC analysis of clinical scores assigned to larvae (*n* = 30 per group, three independent experiments) infected with either WT or ΔHla strain at 1.0 × 10^5^ CFU/larva. Clinical scores for melanization and activity were recorded daily after infection. **(B)** Bacterial burden observed in larval homogenates, expressed as log_10_ CFU/sample. Infected larvae (*n* = 6 per group, two independent experiments) were sacrificed the day after infection. LDV = lower detectable value. **(C)** Survival curves of larvae of *G. mellonella* (*n* = 30 per group, three independent experiments) infected with either WT or ΔHla *S. aureus* USA300 LAC strains at 1.0 × 10^5^ CFU/larva. In **(A,B)**, each single dot reported data for one animal, while the solid red line was the median value of the group. Legend: *p < 0.05. For **(A)**, the Kruskal-Wallis, uncorrected Dunn’s post-test was used to assess significance. For **(B)**, the Mann-Whitney post *t*-test was used. For **(C)**, the log-rank (Mantel-Cox) and Gehan-Breslow-Wilcoxon tests were used.

Next, we evaluated whether Hla increased *S. aureus* virulence during an active infection. To assess Hla’s contribution to the virulence of *S. aureus* in larvae of *G. mellonella*, we first established an infection using the methicillin-resistant *S. aureus* (MRSA) USA300 LAC strain. Larvae were infected with increasing bacterial doses, and clinical signs, CFU counts (at day one post-infection), and survival were monitored. All readouts were dose-dependent (Figure 1). A dose of 1.0 × 10^5^ CFU/larva was selected for subsequent studies because: (i) most of the animals developed signs of disease; (ii) bacteria could be counted in all larvae on the day after infection with acceptable variability; and (iii) lethality at day 3 post-infection was around 75% (LD75). Using this infective dose, we assessed the impact of the Hla toxin in this infection model by comparing the infectivity of the *S. aureus* USA300 LAC WT strain with that of the ΔHla strain. Although the *in vitro* growth of the ΔHla derivative was almost identical to that of the WT strain (data not shown), *G. mellonella* infected with the ΔHla strain showed reduced clinical scores and significantly lower CFU counts, as well as higher survival rates compared to the WT strain, showing that Hla could enhance USA300 virulence in this infection model (Figure 2).

### The *G. mellonella* invertebrate model of Hla toxicity is suitable for assessing the inhibitory properties of anti-Hla human mAbs

Having demonstrated that *G. mellonella* was suitable for detecting the pathogenic effect of *S. aureus* Hla, we wondered whether a specific anti-Hla treatment could also prevent toxicity in this *in vivo* model. For these experiments, we generated an equivalent mAb to Suvratoxumab, a well-known anti-Hla human mAb that has already been assessed in clinical trials (Yu et al., 2017; François et al., 2021). Before performing neutralization experiments, we evaluated the toxicity of this mAb on its own, demonstrating no apparent toxicity up to 7 days post-injection with a dose of 74 ng/larva of anti-Hla mAb (7.4 mg/mL solution, 10 μL injection—data not shown).

**FIGURE 3:**
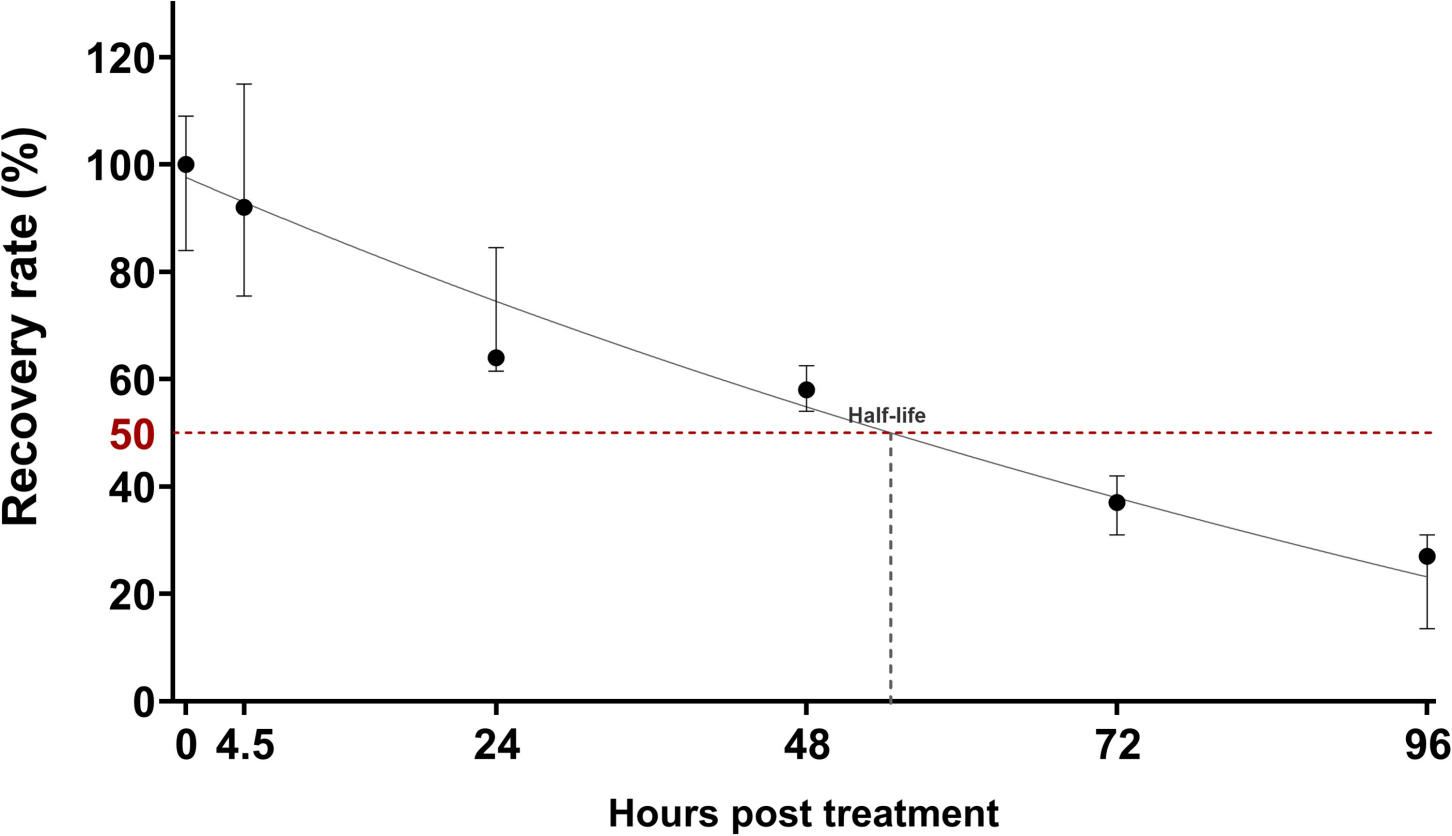
Anti-Hla human IgG1 monoclonal antibody stability *in vivo* in larvae of *G. mellonella* during the time. Data were expressed as percentage of Luminex titer during the time as compared to the median value obtained in a control group (*n* = 5) at time 0 (larvae treated with the mAb and immediately sacrificed). Black dots reported the median value of the group, while the bars were the 25 and 75 percentiles. The horizontal red line depicted the 50% value and was used to visually show the half-life of the mAb. Five larvae/timepoint were sacrificed to perform the analysis.

Next, we assessed the stability of the mAb in larvae by measuring its activity in larval homogenates—evaluated as its ability to recognize the antigen in a Luminex-based assay—at different times post-injection. A slight loss of mAb activity (8%) was observed beginning at 4.5 h post-injection, with an estimated half-life of 51.7 h post-injection (Figure 3).

**FIGURE 4:**
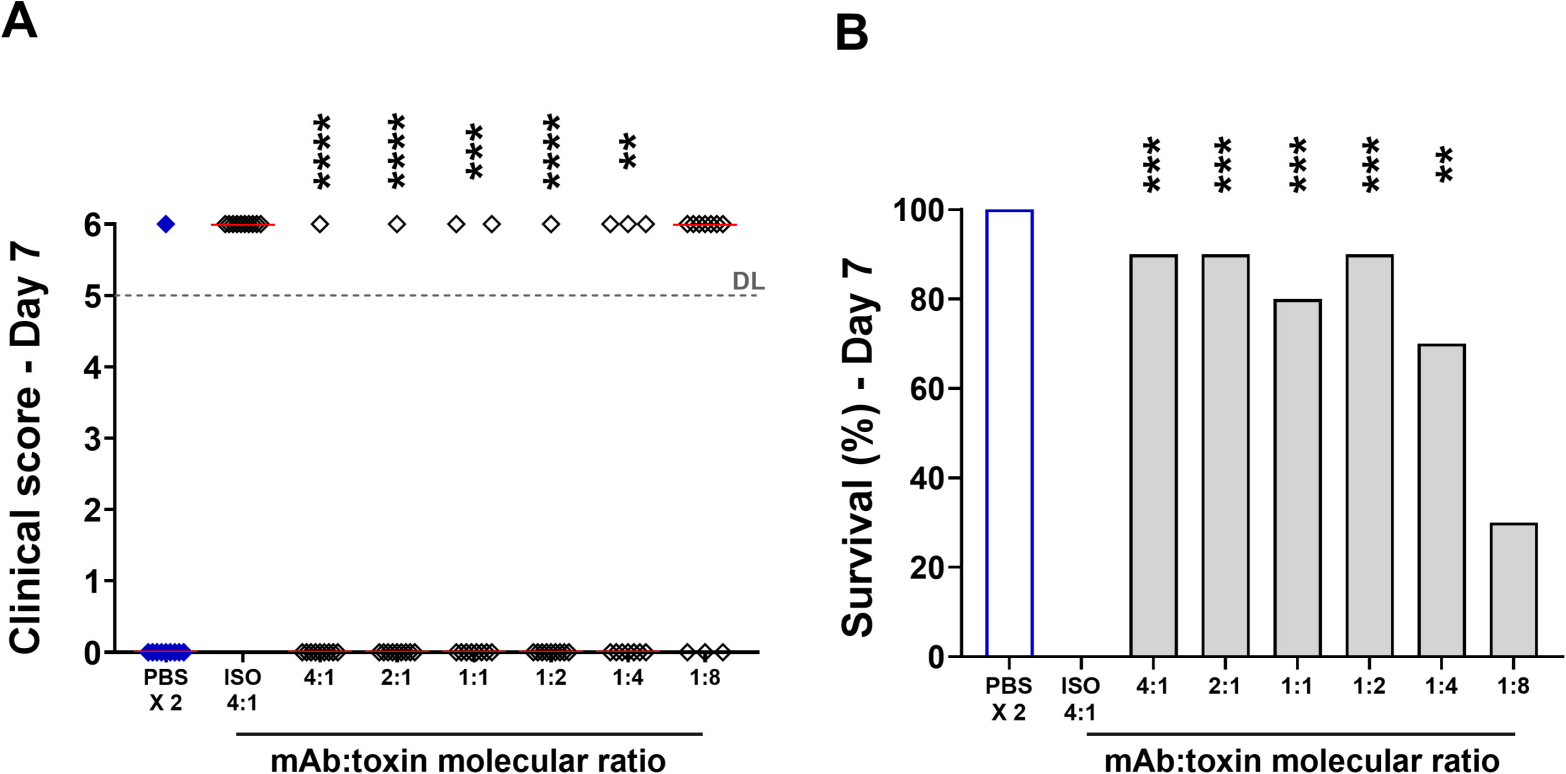
An anti-Hla monoclonal antibody inhibited Hla toxicity *in vivo* in larvae of *G. mellonella*. Signs and survival of larvae at day 7 after Hla intrahemocoelic injection. **(A)** Clinical scores of *G. mellonella* larvae injected with Hla measured at day 7 post-treatment. Single dots reported data from one animal, while the red line was the median value of the group. Dead animals were scored with a “6” and were reported above the grey dotted Dead Larvae (DL) line. **(B)** Percentage of survived larvae at day 7 post-injection. Columns reported cumulative data. The X-axis indicates the antibody:toxin molar ratio for each group. For both graphs, each group was composed by 10 larvae, two independent experiments have been performed, the “PBS 2X” group, in which animals were treated twice with PBS, was used as control group and the “ISO 4:1” was the isotype control group where the mAb was used at a 4:1 molar ratio as respect to the Hla toxin. Legend: **p < 0.01, ***p < 0.001, ****p < 0.0001, computed with comparison between anti-Hla treated larvae and isotype control (ISO) treated larvae. For **(A)**, the Kruskal-Wallis, uncorrected Dunn’s post-test was used to assess significance. For **(B)** the Fisher’s exact test was used to assess significance.

Based on these results, to maximize the potential beneficial effect of the treatment, we decided to pretreat larvae with the mAb at 7.4 mg/mL 4 h before Hla injection. The mAb was able to almost fully neutralize Hla toxicity *in vivo* for at least 7 days after toxin treatment, even at a molar ratio of one mAb molecule to four toxin molecules, confirming results reported in the literature in mouse models (Tkaczyk et al., 2012; Hua et al., 2015; Tkaczyk et al., 2018; Figure 4).

### Blocking Hla toxicity *in vivo* reduces the virulence of the USA300 LAC *S. aureus* strain

**FIGURE 5:**
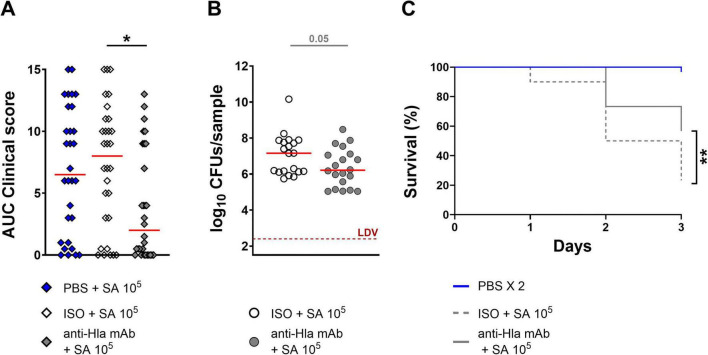
The anti-Hla monoclonal antibody is able to partially protect larvae of *G. mellonella* against a lethal infection with the *S. aureus* USA300 LAC strain. **(A)** AUC analysis of clinical scores assigned to larvae (*n* = 30 per group, three independent experiments) pre-treated with PBS, an ISO or the anti-Hla mAb and then infected with *S. aureus* USA300 LAC strain at 1.0 × 10^5^ CFU/larva. Clinical scores for melanization and activity were recorded for three days after infection. **(B)** Bacterial burden observed in larval homogenates, expressed as log_10_ of CFUs/sample. Infected larvae (*n* = 20 per group, two independent experiments) were sacrificed the day after infection. LDV = lower detectable value. **(C)** Survival curves of larvae of *G. mellonella* (*n* = 30 per group, three independent experiments) pretreated with ISO or the anti-Hla mAb and then infected with the S. aureus USA300 LAC strain. The “PBS 2X” group, in which animals were treated twice with PBS, was used as control group. For **(A)** and **(B)**, each single dot reported data for one animal, while the solid red line was the median value of the group. Legend: *p < 0.05, **p < 0.01. For **(A)** and **(B)**, the Mann-Whitney post *t* test was used. For **(C)**, the log-rank (Mantel-Cox) and Gehan-Breslow-Wilcoxon tests were used.

**FIGURE 6:**
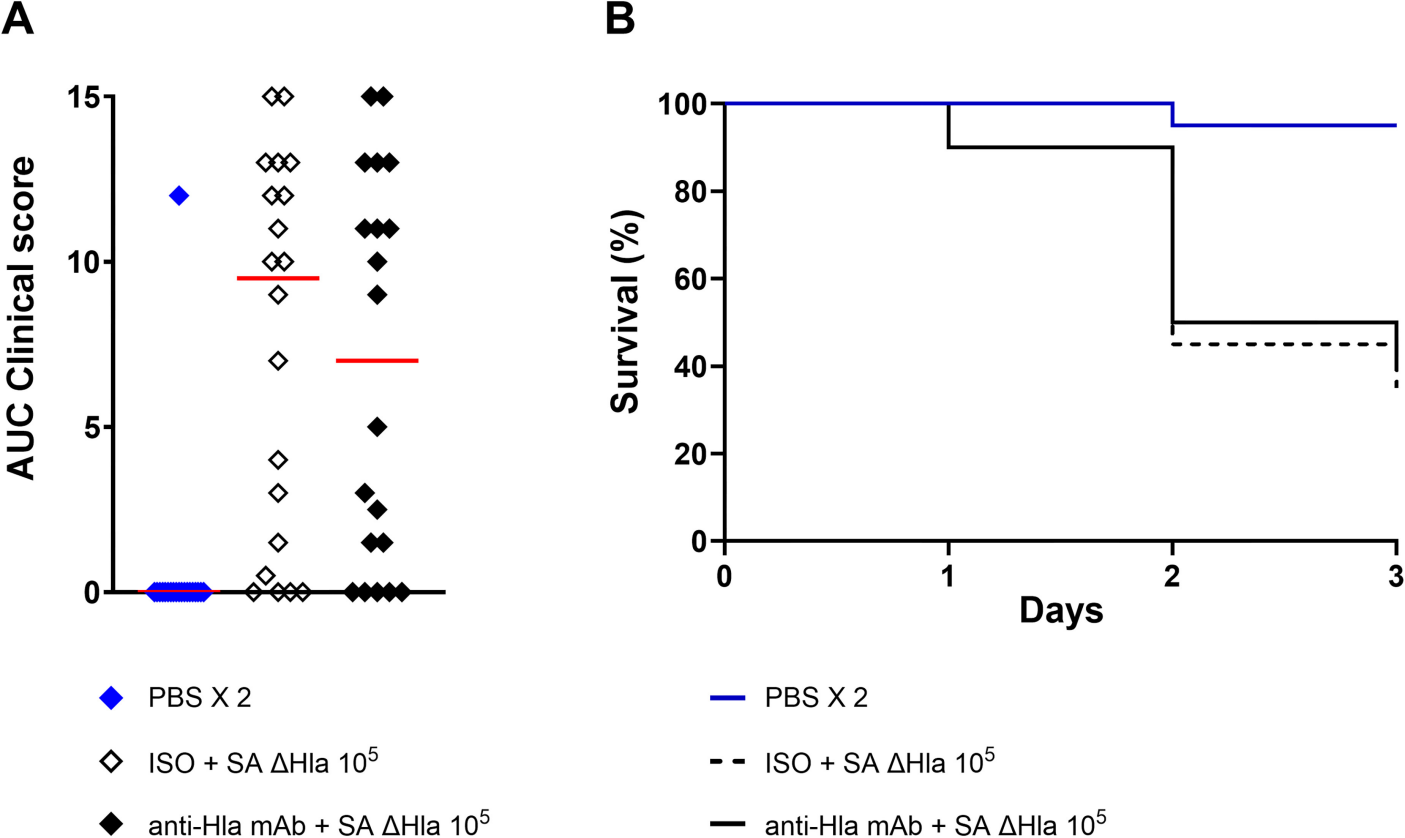
Anti-Hla *in vivo* efficacy is specific for *S. aureus* producing Hla toxin. The tested mAb against the Hla was not able to protect larvae when a ΔHla *S. aureus* USA300 LAC strain was used in the infection experiments. **(A)** AUC analysis of clinical scores assigned to larvae (*n* = 20 per group, two independent experiments) pretreated with an ISO or the anti-Hla mAb and then infected with the ΔHla *S. aureus* USA300 LAC at 1.0 × 10^5^ CFU/larva. Clinical scores for melanization and activity were recorded up to 3 days after infection. Each single dot reports data for a single animal, while the red line is the median value of the group. **(B)** Survival curves of larvae of *G. mellonella* pretreated with ISO or the anti-Hla mAb and then infected with *S. aureus* ΔHla. The “PBS 2X” group, in which animals were treated twice with PBS, was used as control group.

To finally determine whether the model was also suitable for assessing the anti-Hla properties of a human mAb during *S. aureus* infection, larvae were pre-treated with the mAb, an isotype control (or PBS), and were subsequently infected (4 h ± 30 min later) with a lethal dose of *S. aureus* USA300 LAC. Larvae treated with the anti-Hla mAb exhibited significantly reduced signs compared to those receiving the isotype control and also showed a decrease in CFU counts (Figures 5A,B). Finally, survival was significantly increased in larvae treated with the anti-Hla mAb compared to the isotype control group, rising from approximately 20% to around 50% (Figure 5C). Importantly, the mAb had no effect when larvae were infected with the ΔHla strain (Figure 6), demonstrating target specificity.

## Discussion

Bacterial infections pose a critical threat to global health, with those caused by antibiotic-resistant bacteria potentially becoming the major cause of mortality within 20–30 years in the high-income countries (Boucher et al., 2009; Cassini et al., 2019). Key contributors to this issue are the ESKAPE-E pathogens (*Enterococcus faecium*, *S. aureus*, *Klebsiella pneumoniae*, *Acinetobacter baumannii*, *Pseudomonas aeruginosa*, *Enterobacter* species, and *Escherichia coli*), which have become the focus of research aimed at developing new preventive and therapeutic strategies (Ayobami et al., 2022; Miller and Arias, 2024).

Animal models of infections have gained particular importance in recent decades for studying host-pathogen interactions and developing new preventive and therapeutic strategies aimed at treating bacterial infections. Several examples exist, with rats and, particularly, mice being the main species used (Gyssens, 2019; Sarkar and Heise, 2019). The use of these species in research, despite their importance, has been strongly criticized both for ethical reasons and for the high costs associated with them. This is the main reason why, in the last twenty years, several alternative infection models such as those employing *Danio rerio*, *Caenorhabditis elegans*, *Drosophila melanogaster*, and *G. mellonella* have been developed (Apidianakis and Rahme, 2011; Tsai et al., 2016; Brassinga and Sifri, 2019; Kaito et al., 2020; Pereira et al., 2020; Nayak and Mishra, 2022; Ravindran and Mukherjee, 2025). Among these, *G. mellonella* has yielded very promising results and has become one of the most widely used alternative animal model for *in vivo* studies in recent years, with more than 1,100 publications per year during the 3-year period 2022-2024 (Pereira et al., 2020; Serrano et al., 2023). Relatively easy to handle, inexpensive, and possessing a simple yet fully functional innate immune system, *G. mellonella* has been recognized by the scientific community as an essential tool for studying the mechanisms that enhance bacterial virulence and for proposing new therapeutic interventions, such as antibiotics (Tsai et al., 2016; Ménard et al., 2021). More specifically, *G. mellonella* possesses a sophisticated innate immune repertoire (hemocyte-mediated phagocytosis, melanization, antimicrobial peptides and related humoral responses) that is functionally analogous in several aspects to mammalian innate immunity, supporting its use for early-stage, ethically favorable *in vivo* screening of anti-infectives and anti-virulence candidates (Asai et al., 2023). Despite the extensive literature in the field, we found that no study had been dedicated to validating this model for characterizing virulence factors and testing specific interventions aimed at blocking these factors. The objective of this work was, therefore, to evaluate the usability of this model in preclinical studies aimed at finding new anti-virulence factors treatments. For this first proof-of-concept study, we selected the ESKAPE-E pathogen *S. aureus* and its secreted toxin Hla as the virulence factor to be investigated. Several reasons supported these choices: (i) numerous papers have been published on the ability of this pathogen to infect larvae; (ii) Hla has been demonstrated to be an important virulence factor in mouse models of skin infection, sepsis, and pneumonia; (iii) a few studies indicated that the downregulation of Hla (together with other virulence factors) mediated by chemicals negatively impacted *S. aureus* virulence in larvae of *G. mellonella* (Silva et al., 2017; Xu et al., 2025); and (iv) specific anti-Hla interventions have been developed and protect mice against *S. aureus* infections.

While *in silico* analysis identified a protein in *G. mellonella* with approximately 41% identity to human ADAM10, the recognized receptor for Hla, this finding serves as supportive context rather than a central focus of this study (see Supplementary Figure 1). The observed toxicity of Hla in *G. mellonella* larvae and its role in enhancing *S. aureus* virulence were robustly demonstrated through experimental approaches detailed in this manuscript. However, the role of ADAM10 homology in mediating Hla binding and toxicity was not functionally validated via inhibition or blocking assays. Future studies could explore this potential mechanistic relationship in more depth. The current findings provide a proof-of-principle that *G. mellonella* serves as a reliable and ethically favorable model for studying Hla-mediated virulence and testing anti-Hla therapeutic interventions. When administered to larvae, Hla was indeed able to kill the animals in a dose-dependent manner, and only when the toxin was fully active (Table 2). Interestingly, Hla was also clearly able to enhance the virulence of a MRSA USA300 strain in a model mimicking mouse sepsis, further underlining the importance of this virulence factor for the bacterium (Figures 1, 2).

*G. mellonella* was, therefore, a suitable model for studying the virulence mechanisms of specific bacterial antigens. However, to validate the model and render it suitable for testing the efficacy of specific anti-virulence factor treatments, we needed to determine whether one of these antibacterial treatments could protect larvae against Hla toxicity and, ideally, against *S. aureus* infection.

MAbs have been primarily used as anti-tumoral and anti-inflammatory treatments, and more recently also to treat infectious diseases. Clinical trials for assessing safety, pharmacokinetics and efficacy of different anti-Hla mAbs against *S. aureus* ventilator-associated pneumonia have been performed (Magyarics et al., 2019). MAbs were shown to be safe and with a sufficient half-life, but unfortunately they did not reach the primary efficacy endpoints. Before being tested in humans, they have proven highly efficacious in preclinical host models (Tkaczyk et al., 2012; Hua et al., 2015; Tkaczyk et al., 2018). Therefore, we were interested in evaluating whether *G. mellonella* models have yielded similar results, suggesting that they could potentially replace mouse models for a more ethical and inexpensive approach. The human IgG1 anti-Hla Suvratoxumab was selected to prove this hypothesis (François et al., 2021).

Firstly, we showed that the human mAb was not toxic to *G. mellonella* and we quantified mAb levels in whole-larva homogenates over time after administration, to estimate in-larva stability. Even if *G. mellonella* does not recapitulate mammalian pharmacokinetics (insects lack liver and kidneys and rely on insect-specific physiology), we were able to observe that a single anti-Hla mAb dose resulted in only ∼8% loss of detectable antibody at 4.5 h (the interval prior to Hla challenge or *S. aureus* infection) and an apparent post-injection half-life of approximately two days, indicating that antibody levels were largely preserved during the protection window (Figure 3).

When administered before either Hla injection or *S. aureus* infection, this anti-Hla specific treatment was able to block Hla-driven toxicity and protect the host (Figures 4, 5). These experiments clearly demonstrated that the *G. mellonella* model not only yielded comparable results in terms of Hla toxicity and its enhancement of *S. aureus* virulence, but also in identifying specific anti-Hla treatments closely mimicking the results obtained in mice.

The results obtained in the present work open up the possibility of using the *G. mellonella in vivo* model as a high-throughput, cost-effective natural platform for testing novel specific anti-virulence factor treatments such as mAbs. Infection models leveraging larvae of *G. mellonella* have already been established for bacteria, viruses, and fungi, highlighting the high potential of this species in the field (Tsai et al., 2016; Silva et al., 2017; Borman, 2018; Asai et al., 2019; Pereira et al., 2020; Durieux et al., 2021; Menard et al., 2021; Ménard et al., 2021; Serrano et al., 2023; Marena et al., 2025). Interestingly, in this work we focused on bacterial secreted toxins, but the efficacy of a novel mAb against a non-secreted virulence factor of *Candida albicans* was also recently reported, suggesting this *in vivo* model could be applied also to different kinds of virulence factors (Antoran et al., 2020). However, because therapeutic monoclonal antibodies in mammals may also exert benefit through immune-mediated mechanisms (beyond antigen binding/neutralization) that involve adaptive immunity and mammalian-specific effector pathways, confirmation of efficacy and mechanism in vertebrate models (e.g., mice) remains essential before positioning *G. mellonella* as a stand-alone preclinical system for antibody therapeutics. At present, it remains too early to consider *G. mellonella* as the sole or fully translatable model for predicting human clinical outcomes. The predictive value of alternative invertebrate systems for human infection and therapy is still being defined and requires broader validation across pathogens, virulence factors, and treatment modalities. Nevertheless, our findings provide a first proof of concept indicating that this model can capture biologically meaningful, toxin-driven pathogenic mechanisms *in vivo*. In this context, *G. mellonella* may serve as a valuable bridge between traditional *in vitro* assays and more comprehensive *in vivo* approaches, offering the potential to support early screening of targeted antimicrobial and anti-virulence strategies, including the ability, for selected readouts, to discriminate between less and more potent therapeutic interventions against resistant pathogens.

## Data Availability

The original contributions presented in this study are included in the article/Supplementary material, further inquiries can be directed to the corresponding author.
