# Supplementary material for: Galleria mellonella model recapitulates Staphylococcus aureus hemolysin toxicity and antibody-mediated mechanisms of protection
Source: Front Microbiol. 2026 May 14;17:1809973. doi: 10.3389/fmicb.2026.1809973 (PMC13215939; doi:10.3389/fmicb.2026.1809973)
Supplement: Supplementary file 1 [file Supplementary_file_1.docx]

# Supplementary Material

## Figure S1. ADAM10 protein blasting between *H. sapiens* and *G. mellonella* species.

The BLAST alignment between the human ADAM10^1^ and the *G. mellonella* “exposed disintegrin and metalloproteinase domain-containing protein 10 homologue” (XP_026755746.1) revealed 41% of identities and 55% of positives. In the alignment below, the metalloprotease active site and the disintegrin loop are highlighted in yellow and blue, respectively.

ADAM10 -------------MVLLRVLILLLSWAAGMGGQYGNPLNKYIRHYEGLSYNVDSLHQKHQ 47

XP_026755746.1 MKSVCLEATMFSEIGYIVFLLLALPYIESASRLTNTRLSEYIEHYEPLDYDADAIHEQHL 60

: : .*:* * : . . .. *.:**.*** *.*:.*::*::*

ADAM10 RAKRAVSHEDQFLRLDFHAHGRHFNLRMKRDTSLFSDEFKVETSN-KVLDYDTSHIYTGH 106

XP_026755746.1 RRRRSTD-TQPDLRLHFRAHGRRFNLRLRRDLSAFSDDFKVEGSQGQLHDVDTSHIYHGE 119

* :*:.. : ***.*:****:****::** * ***:**** *: :: * ****** *.

ADAM10 IYGEEGSFSHGSVIDGRFEGFIQTRGGTFYVEPAERYIKDR--TLPFHSVIYHEDDINYP 164

XP_026755746.1 LADEPHSTVFGSVTEGVFEGKIMSKDGAFYVEHARRYFPPNGTRTRVHSVIYKESDVSDP 179

: .* * .*** :* *** * ::.*:**** *.**: . .*****:*.*:. *

ADAM10 HK---YGPQGGCADH-SVFERMRKYQMTGVEEVTQIPQ---------------------- 198

XP_026755746.1 YAHRRHGHVGGCGITDEVVQWMERIQNSGVDDDPPTSPTTTPPPSASPPHHTSSPHHIPG 239

: :* ***. .*.: *.: * :**::

ADAM10 ------------------------EEHAANGPELLRKKRTTSAEKNTCQLYIQTDHLFFK 234

XP_026755746.1 SSPPHPNSLRDDPPRHWDYPHHNKYSRSANTGEHSRTRRATLDNRNTCSLYIQTDPLIWR 299

.::** * *.:*:* ::***.****** *:::

ADAM10 YY------------------GTREAVIAQISSHVKAIDTIYQTTDFSGI---RNISFMVK 273

XP_026755746.1 HVREGFPEHRDPTKRTEVDMKTREEILSLISHHVTAVNYIYRDTKFDGRMVHRNIKFEVQ 359

: *** ::: ** **.*:: **: *.*.* ***.* *:

ADAM10 RIRINTTA---DEKDPTNPFRFPNIGVEKFLELNSEQNHDDYCLAYVFTDRDFDDGVLGL 330

XP_026755746.1 RIKIDDDSFCVTHQYDRNQFCHENIDVSNFLNLHSLGNHEDFCLAYVFTYRDFTGGTLGL 419

**:*: : .: * * . **.*.:**:*:* **:*:******* *** .*.***

ADAM10 AWVGAPSGSSGGICEKSKLYSD-------GKKKSLNTGIITVQNYGSHVPPKVSHITFAH 383

XP_026755746.1 AWVASASGASGGICEKYKTYTETIGGMYQSTKRSLNTGIITFVNYNSRVPPKVSQLTLAH 479

***.: **:******* * *:: ..*:********. **.*:******::*:**

ADAM10 EVGHNFGSPHDSGTECTPGESKNLGQKENGNYIMYARATSGDKLNNNKFSLCSIRNISQV 443

XP_026755746.1 EIGHNFGSPHDYPSECRPGG-------QQGNFIMFASATSGDRPNNSKFSTCSVGNISAV 532

*:********* :** ** ::**:**:* *****: **.*** **: *** *

ADAM10 LEK----KRNNCFVESGQPICGNGMVEQGEECDCGYSD-QCKDECCFDANQ--------P 490

XP_026755746.1 LDAVRDGRKRNCLTASAGAFCGNKIVEDGEECDCGYDENECRDHCCYPRQVSSYDKERNS 592

*: ::.**:. *. :*** :**:********.: :*:*.**: :

ADAM10 EGRKCKLKPGKQCSPSQGPCCTAQC---AFKSKSEKCRDDSDCAREGICNGFTALCPASD 547

XP_026755746.1 TAKGCTRKANTQCSPSQGPCCHARTCQFVLAFRNQTCREATECSHASVCSGRSAECPEPR 652

.: *. * ..********** *: .: :.:.**: ::*:: .:*.* :* **

ADAM10 PKPNFTDCNRHTQVCINGQCAGSICEKYGLEECTCASSDG---------KDDKELCHVCC 598

XP_026755746.1 VMSNHTKCNNGTQLCNAGECTGSICLAWNMKECFLSSAPQRLGDGVTAVVDRRALCQLAC 712

*.*.**. **:* *:*:**** :.::** :*: * : **::.*

ADAM10 MKKMDPSTCASTGSVQWSRHFSGRTITLQPGSPCNDFRGYCDVFMRCRLVDADGPLARLK 658

XP_026755746.1 QTGPEPDSCQSTADFARRVGLPPGGISLRPGSPCDNFQGYCDVFLKCRAVDAEGPLARLK 772

. :*.:* **... : *:*:*****::*:******::** ***:*******

ADAM10 KAIFSPELYENIAEWIVAHWWAVLLMGIALIMLMAGFIKICSVHTPSSNPKLPPPKPLPG 718

XP_026755746.1 NLLLNRATLQSVQAWVTEQWWAVLLGGVALVVCMGAFVKCCAVHTPSSNPKRPPARRLSE 832

: ::. :.: *:. :****** *:**:: *..*:* *:********* ** : *

ADAM10 TLKRRRPPQP-IQQPQRQRPRESYQMGHMRR----------------------------- 748

XP_026755746.1 TLRRPMNTLRRMRHPGHAA-ARAPRPGHRRHRPSKGDYSAPVAYQARDIASAPPGPSGRR 891

**:* :::* : .: : ** *:

ADAM10 -------------------- 748

XP_026755746.1 GEPYATAYPQSYEMRPPQKV 911

## Figure S2. Gentamicin completely abrogates *S. aureus* lethality.

Pre-treatment of larvae of *G. mellonella* with gentamicin (25 mg/mL) completely protects larvae from a lethal dose of *S. aureus* USA300 LAC (1×10^7^ CFUs/larva, LD100). **(A)** Clinical scores of *G. mellonella* larvae injected and monitored until 24 hours post treatment. Single dots report data for one animal and the red line is the median value of the group. Dead animals were scored with a “6” and related data were reported above the grey dotted Dead Larvae (DL) line. **(B)** Survival curves of larvae (*n* = 10 per group) of *G. mellonella* at 16, 18 and 24 hours post treatment. Legend: *****p* < 0.0001. For **(A)**, the Kruskal-Wallis, uncorrected Dunn’s post-test was used to assess significance among groups. For **(B)**, the log-rank (Mantel-Cox) and Gehan-Breslow-Wilcoxon tests were used to assess significance respect to mock-treated larvae (PBS X 2).


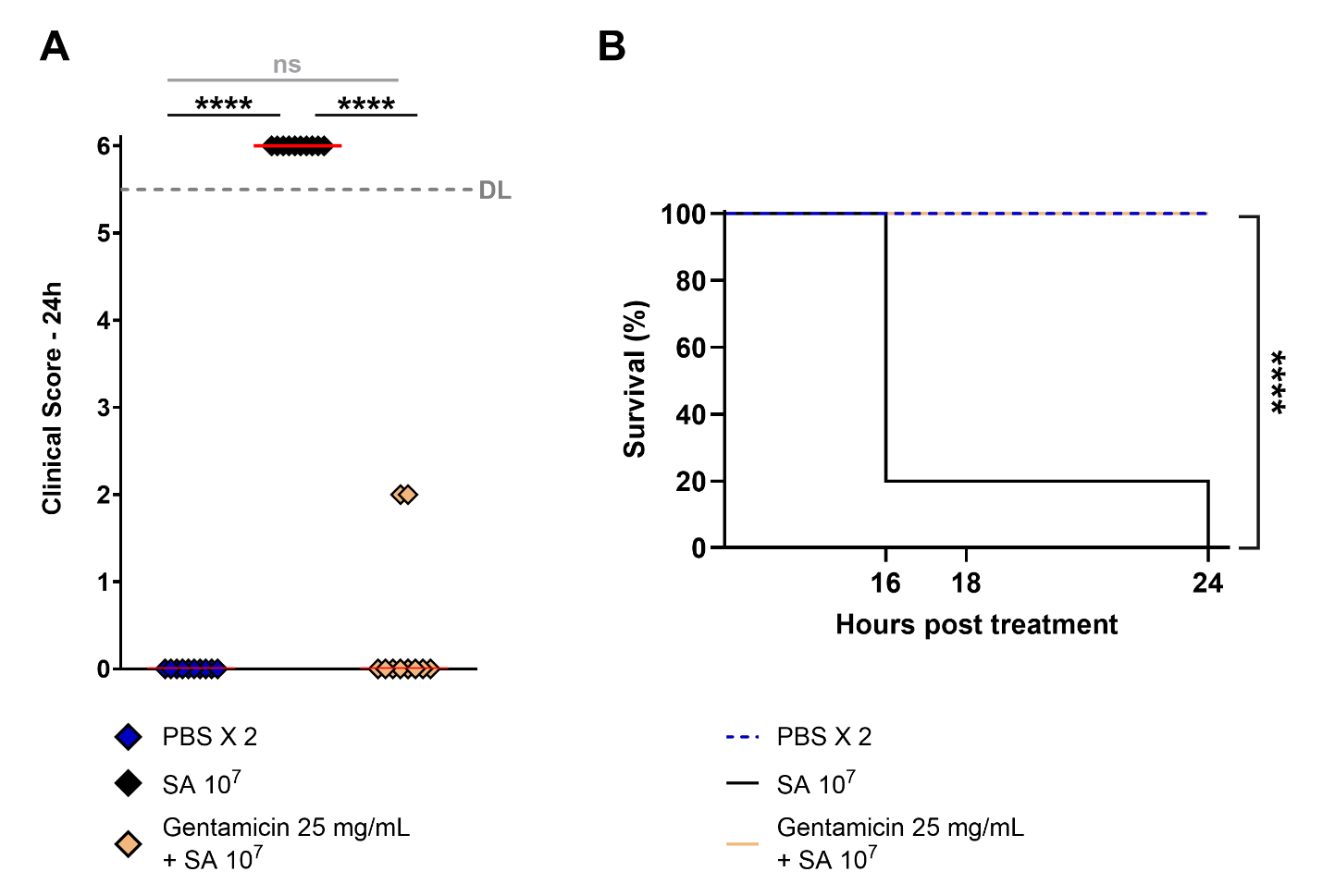


^1^Wolfsberg T. G. et al. (1995). ADAM, a novel family of membrane proteins containing A Disintegrin And Metalloprotease domain: multipotential functions in cell-cell and cell-matrix interactions. *J. Cell Biol.* 131, 275-278. doi: [10.1083/jcb.131.2.275](https://doi.org/10.1083/jcb.131.2.275)
